# Stress-stimulated epinephrine induces premature senescence in dermal fibroblasts and contributes to impaired skin wound healing

**DOI:** 10.1590/1414-431X2025e14472

**Published:** 2025-06-16

**Authors:** J.T. da Silva, C.O.S. Meira, J.S. Nogueira, M. Lanzetti, B. Romana-Souza

**Affiliations:** 1 Departamento de Histologia e Embriologia, Universidade do Estado do Rio de Janeiro, Rio de Janeiro, RJ, Brasil; 2 Laboratório de Histocompatibilidade e Criopreservação, Universidade do Estado do Rio de Janeiro, Rio de Janeiro, RJ, Brasil; 3 Instituto de Ciências Biomédicas, Universidade Federal do Rio de Janeiro, Rio de Janeiro, RJ, Brasil

**Keywords:** Psychological stress, Aging, Skin wound healing, Cellular senescence, Dermal fibroblasts

## Abstract

External factors accelerate cellular senescence in the skin and compromise its repair process. Psychological stress impairs skin wound healing, but no study examined its role in cellular senescence during skin tissue repair. Thus, this study evaluated the effect of stress on cellular senescence during skin wound healing. Human dermal fibroblasts (HDFs) and human skin from aged and young donors were treated with a non-lethal dose of epinephrine or hydrogen peroxide following a stress-induced premature senescence protocol. *In vitro*, epinephrine or hydrogen peroxide promoted the expression of senescence-associated β-galactosidase, p53, and notch target gene hairy and enhancer of split 1 (HES1) in HDFs, confirming the induction of premature senescence. A higher expression of matrix metalloproteinase-9 and interleukin-8 was observed in HDFs incubated with epinephrine or hydrogen peroxide, confirming a pro-inflammatory senescence-associated secretory phenotype. The protein levels of silent information regulator sirtuin 1, which is associated with a longer lifespan, were not changed in HDFs submitted to stressful conditions. In *ex vivo* experiments, epinephrine administration impaired wound closure and increased HES1 expression in aged human skin. In conclusion, stress-induced high epinephrine level induced premature senescence in HDFs, which contributes to impaired wound healing in young and aged skin.

## Introduction

Aging is the natural process of degeneration of the body that depends on time and leads to the physiological decline of organs. The proportion of the world population over the age of 60 years is growing at an increasing rate, and it is expected that it will nearly double from 12 to 22% in one decade (1). This demographic shift will directly impact the demands for specialized health services and the cost of treatments in geriatric medicine. Skin wound healing is a dynamic process of closure of an open area of skin, reducing the chances of infection. In elderly humans, skin wound healing is naturally less efficient due to the decreased functioning of many cell types and pathways involved in this process (2). Therefore, in individuals over the age of 65, the development of chronic wounds (such as cancer wounds or pressure ulcers) is associated with a three times higher probability of mortality (2,3).

Dermal fibroblasts contribute to skin wound healing by depositing a matrix rich in collagen and remodeling the tissue (4). Several fibroblasts also differentiate into myofibroblasts that contract the edges of skin wounds (4). During skin aging, most dermal fibroblasts develop a senescent phenotype characterized by irreversible proliferation arrest and increased release of a senescence-associated secretory phenotype (SASP) (5). Senescent fibroblasts are characterized by increased expression of metalloproteinase-9 (MMP-9), notch target gene hairy and enhancer of split 1 (HES1), which is a biomarker of notch activation, and interleukin-8 (IL-8), a pro-inflammatory cytokine (5,6). Moreover, a reduction of silent information regulator sirtuin 1 (SIRT1) protein expression, which is associated with the maintenance of longevity, can be observed in senescent cells (7). In skin wounds, SASP fibroblasts induce chronic inflammation and increase the degradation of the collagen matrix by matrix metalloproteinase secretion, which may lead to the formation of chronic wounds (8). Several extrinsic factors, such as oxidative stress or ultraviolet irradiation, can accelerate the development of a senescent phenotype in dermal fibroblasts (9). Repeated psychological stress is another possible contributing factor of aging that leads to the acceleration of the shortening of the telomere and signs of skin aging in humans and mice (10-14). These stress-induced alterations are associated with excessive secretion of glucocorticoids and catecholamines (epinephrine and norepinephrine) through the activation of hypothalamic-pituitary-adrenocortical and sympathetic-adrenal axes (15). In young mice, repeated stress compromises the closure of skin wounds mainly by prolonged inflammation (16). However, no study has yet demonstrated if repeated stress alters skin healing through the induction of premature senescence in fibroblasts. Thus, the aim of this study was to investigate the effect of repeated stress on dermal fibroblast senescence during skin wound healing using *ex vivo* and *in vitro* models.

## Material and Methods

### Cell culture

Normal human dermal fibroblast cell line HFF-1 from foreskin (Banco de Células do Rio de Janeiro, Xerém, Brazil, catalogue number: 0275) were grown in Dulbecco's modified Eagle medium (DMEM) (Life Technologies Corp., USA) supplemented with 15% of fetal bovine serum (FBS) (Cultilab, Brazil), 1 mM of sodium pyruvate (Sigma-Aldrich, Inc., USA), 100 units/mL of penicillin, 100 μg/mL of streptomycin, and 0.25 μg/mL of amphotericin A (Nova Biotecnologia, Brazil). Cells were maintained in an atmosphere with 5% CO_2_ and a temperature at 37°C. Cells were used at 85-90% of confluence and passage 6-7.

### *Ex vivo* human skin model

In this experiment, human skin samples were obtained from 8 younger participants (age: 10.0±4.2 years; 2 men/6 women with age between 7 and 20 years) and 8 older participants (age: 59.1±5.4 years; 2 men/6 women with age between 53 and 67 years) who were undergoing otoplasty or blepharoplasty. Explants of 6 mm normal human skin were cultured in DMEM (Life Technologies Corp.) with 10% of FBS (Cultilab), 100 units/mL of penicillin, 100 μg/mL of streptomycin, and 0.25 μg/mL of amphotericin A (Nova Biotecnologia). Skin explants were maintained in 5% of CO_2_ at a temperature of 37°C. After 24 h, one full-thickness wound with 2 mm in diameter was created in the center of each explant using a punch biopsy. The epidermis of each explant was not submerged in the media. This experiment was approved by the Ethics Committee for Human Studies from the University of Grande Rio (CAAE: 46799215.1.0000.5283). All patients agreed to participate in the study and signed the consent form. Exclusion criteria were smoking, diabetes, hypertension, obesity, and cutaneous diseases.

### Stress-induced premature senescence (SIPS) model

For induction of SIPS, cells (35,000 cells per well) or explants were treated with 100 μM H_2_O_2_ (Sigma-Aldrich, Inc.) or 100 µM epinephrine hemitartrate (Hypofarma, Brazil) dissolved in medium for one hour per day, followed by a medium change. The treatment with stressors was performed on 4 consecutive days, followed by 2 days of recovery, then 5 more consecutive days of stress treatment (17). The control group received the medium only. The cells were harvested on the last day of treatment. The explants were harvested on the first (day of wounding) and last (10 days after wounding) day of treatment and fixed in 10% formalin at pH 7.2 (Sigma-Aldrich, Inc.) for histological analyses. This experiment was performed two or three times in triplicate. The experimental design is shown in Supplementary Figure S1.

### Cell viability assay

Cell viability was assessed using the MTT (Sigma-Aldrich, Inc.) assay. The cells were incubated with 16 μL of MTT at 5 mg/mL for 4 h at 37°C in atmosphere with 5% CO_2_. Subsequently, the medium was discarded, and the cells were dissolved in 200 μL isopropyl alcohol at 100% (VETEC Química, Brazil). The formazan crystals were read at 570 nm.

### Senescence-associated β-galactosidase (SA-β-gal) staining

For SA-β-gal staining (18), the medium was discarded, and the cells were washed with phosphate-buffered saline (PBS) 1× and fixed with 4% paraformaldehyde in PBS 1× for 1 h at room temperature. After washing with PBS 1×, the cells were incubated with 4 mg/mL 5-bromo-4-chloro-3-indolyl-β-D-galactopyranoside (Sigma-Aldrich, Inc.) in buffer containing 5 mM potassium hexacyanoferrate (III), 5 mM potassium hexacyanoferrate (II) trihydrate, and 2 mM magnesium chloride dissolved in PBS 1× (Sigma-Aldrich, Inc.) for 1 h at 37°C. After washing with PBS 1×, photomicrographs of cells were taken at ×400 magnification using Axio Observer A1 microscope and AxioCam MRc5 camera (Zeiss-Vision, Germany).

### Histological analysis

Formalin-fixed skin samples were processed, embedded in paraffin, and sectioned. The sections (5 μm) were stained with hematoxylin & eosin and digitized using a brightfield digital slide scanner (Pannoramic Midi, 3DHISTECH Ltd., Hungary). The length between the edges of each wound was measured using the image software Pannoramic Viewer (3DHISTECH Ltd.). Results are reported as a percentage of the original wound area on the day of wounding (d0).

### Immunocytochemistry and immunohistochemistry

For immunofluorescence analysis, cells were fixed with 4% paraformaldehyde, permeabilized with 0.5% triton, and blocked with 10% normal goat serum. Subsequently, cells were incubated with antibody against HES1 and an appropriate secondary antibody (Supplementary Table S1). For human skin samples, paraffin-embedded sections were incubated in citrate buffer (pH 6.0) at 70°C. After washing with PBS 1×, sections were blocked with 10% normal goat serum and incubated with antibodies against HES1 and vimentin and appropriate secondary antibodies (Supplementary Table S1). Nuclei were stained using DAPI Fluoromount-G^®^ mounting medium (Southern Biotechnology Associates, Inc., USA). Photomicrographs were taken using a fluorescence microscope (Axio Observer.A1), AxioCam MRc5 camera (Zeiss-Vision), and a 40× objective. Subsequently, the number of HES1-positive cells were counted in the photomicrographs (143,445 mm^2^). For the *in vitro* assay, the results are reported as percentage of HES1-positive cells normalized to number of DAPI-stained nuclei. For human skin samples, the results are reported as the number of HES1-positive cells per mm^2^.

### Flow cytometry

To check the percentage of apoptotic cells, cells were obtained by trypsinization and stained with Annexin V-FITC Apoptosis Detection Kit (#BMS500FI-20; eBioscience, USA), following the manufacturer's instructions. Subsequently, the cells were resuspended in PBS 1× and subjected to flow cytometry (Gallious, Beckman Coulter, Inc., USA).

### Quantitative reverse transcription polymerase chain reaction (qRT-PCR)

Total RNA was extracted from cells and tissue samples using TRizol reagent (Life Technologies Corp.), following the manufacturer's directions. Subsequently, the cDNA of each sample was synthesized using a High-Capacity cDNA Reverse Transcription Kit (Applied Biosystems™, USA). The mRNA levels of p53 were estimated by semi-quantitative PCR on a StepOnePlus Real-Time PCR System (Applied Biosystems™) using Power SYBR Green PCR Master Mix (Sigma-Aldrich, Inc.). Glyceraldehyde‐3‐phosphate dehydrogenase was used as a control. The 2^-ΔΔCt^ method was used to calculate the expression of genes (19). The primers are described in Supplementary Table S2.

### Relative quantification of telomere length by real-time PCR

DNA was extracted from cells using a Biopur Mini Spin Plus kit (Mobius, Brazil), following the manufacturer's directions. The length of telomeres was evaluated by semi-quantitative PCR on a StepOnePlus Real-Time PCR System (Applied Biosystems™) using Power SYBR Green PCR Master Mix (Applied Biosystems™) (20). β-globin was used as a control. For the amplification of telomeres, the following parameters were utilized: an initial denaturation step at 95°C for 10 min, followed by 40 cycles of annealing and extension at 95°C for 15 s and 54°C for 2 min. Concerning the amplification of β-globin, a similar protocol was applied, with an initial denaturation at 95°C for 10 min, followed by 40 cycles of annealing and extension at 95°C for 15 s and 58°C for 1 min. In both instances, the melting curves were generated at a temperature of 60°C. The 2^-ΔΔCt^ method was used to calculate the expression of genes (19). The primers are described in Supplementary Table S2.

### Enzyme-linked immunosorbent assay (ELISA)

The cells were macerated in radioimmunoprecipitation assay buffer (50 mM Tris pH 7.6, 1% Triton X-100, 0.5% sodium dodecyl sulfate, 0.1% sodium deoxycholate, 140 mM sodium chloride, 0.5 mM ethylene glycol-bis (2-aminoethylether)-N, N, N′, N′-tetraacetic acid, and 1 mM ethylenediaminetetraacetic acid) (Sigma-Aldrich, Inc.) with a 1% protease inhibitor cocktail for mammalian cells (Sigma-Aldrich, Inc). The total protein concentration was determined using a commercial bicinchoninic acid protein assay kit (Thermo Fisher Scientific Inc., USA; catalog number: 23225). The protein levels of IL-8 were measured in cell lysates using a commercial ELISA kit (Peprotec, Brazil, catalog number: 900-TM18), following the manufacturer's directions.

### Biochemical analysis

The production of reactive oxygen species (ROS) was estimated in cell lysate using a semi-quantitative colorimetric nitroblue tetrazolium (NBT) assay (21). Briefly, the cell lysate of each sample was combined with 100 μL of NBT 0.1% in PBS 1× and incubated at 37°C for 1 h. After centrifugation (125 *g*, 25°C, 5 min) and PBS 1× washing, the pellet was dissolved in a mixture of potassium hydroxide and dimethyl sulfoxide and read at 620 nm.

### Western blot

The proteins of cell lysates (20 μg) were separated into a 10% polyacrylamide gel containing sodium dodecyl sulfate, transferred to polyvinylidene fluorene membrane (Bio‐Rad Laboratories, USA), and blocked with 5% nonfat skim milk. Subsequently, the membranes were incubated with anti-HES1, anti-MMP-9, anti-SIRT1, proliferating cellular nuclear antigen (PCNA), and an appropriate secondary antibody (Supplementary Table S1). The antigen-antibody complexes were detected using the Immobilon Western kit (Merck Millipore, USA). Bands were measured using ImageJ software (National Institutes of Health, USA) and β-actin was used as a control.

### Statistical analysis

Data are reported as mean and standard deviation. The normality of results was checked using the Shapiro-Wilk and Kolmogorov-Smirnov tests using GraphPad Prism version 10 (GraphPad Software, USA). Comparison of groups was performed using a one-way ANOVA followed by Bonferroni's post-test for parametric data or a Kruskal-Wallis test with Dunn post-test for nonparametric data. The outliers were removed using the ROUT test (Q=1%). P<0.05 was considered statistically significant.

## Results

### High levels of epinephrine induced premature senescence in human dermal fibroblasts

**Figure 1:**
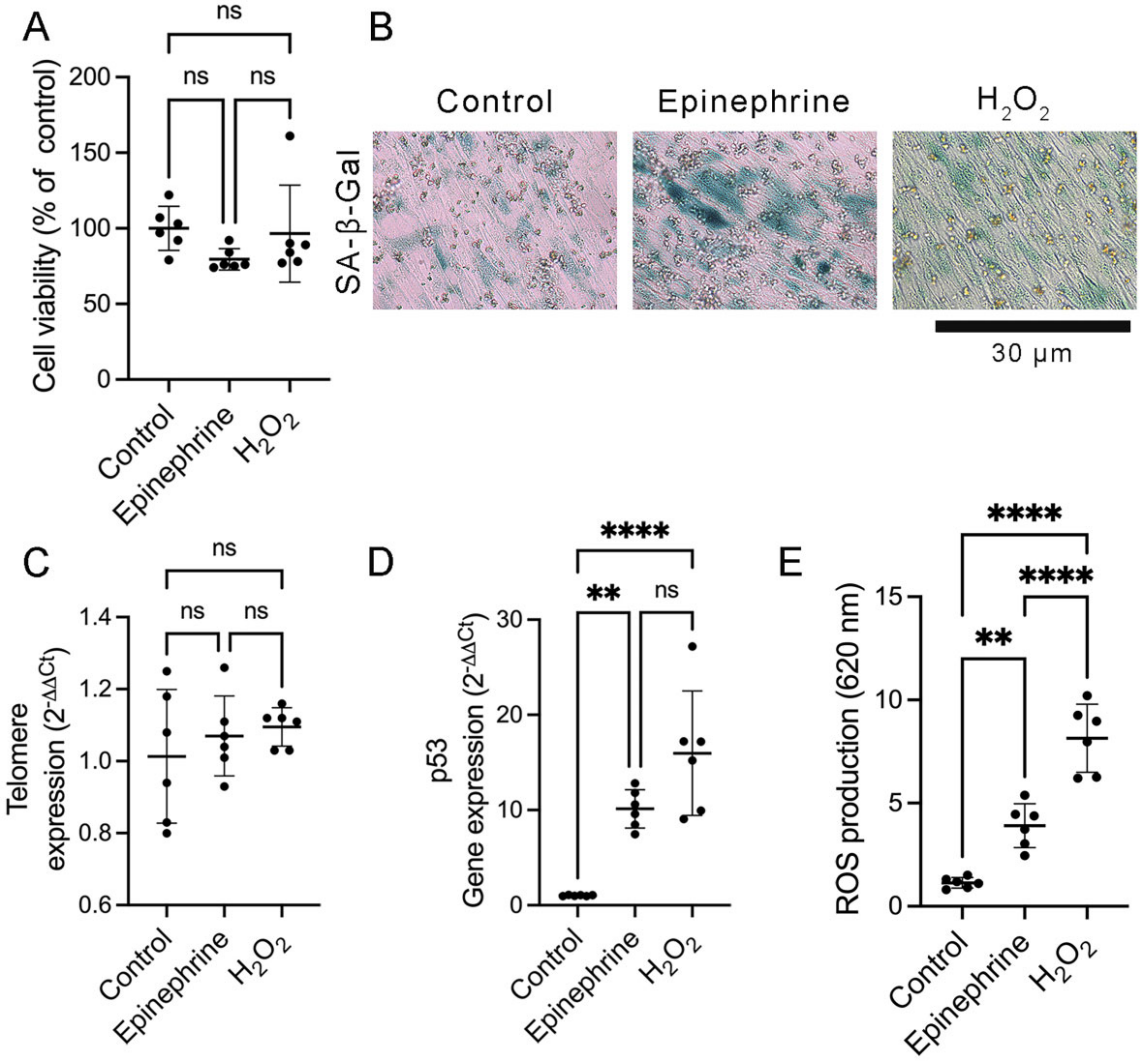
High epinephrine levels induced a premature senescent phenotype in human dermal fibroblasts. **A**, Cell viability (% of control) using 3-(4,5-dimethyl-2-thiazolyl)-2,5-diphenyl-2H-tetrazolium bromide assay. **B**, Representative photomicrographs of senescence-associated β-galactosidase (SA-β-gal) staining. Scale bar=30 µm. **C**, Relative expression of telomere normalized to β-globin detected by quantitative reverse transcription polymerase chain reaction. **D**, mRNA levels of p53 normalized to GAPDH estimated by real-time quantitative reverse transcription polymerase chain reaction. **E**, Reactive oxygen species (ROS) production expressed as absorbance at 620 nm. Data are reported as means±SD (n=6, two independent experiments in triplicate). **P<0.01, ****P<0.0001; one-way ANOVA with Bonferroni's post-test; ns: not significant.

**Figure 2:**
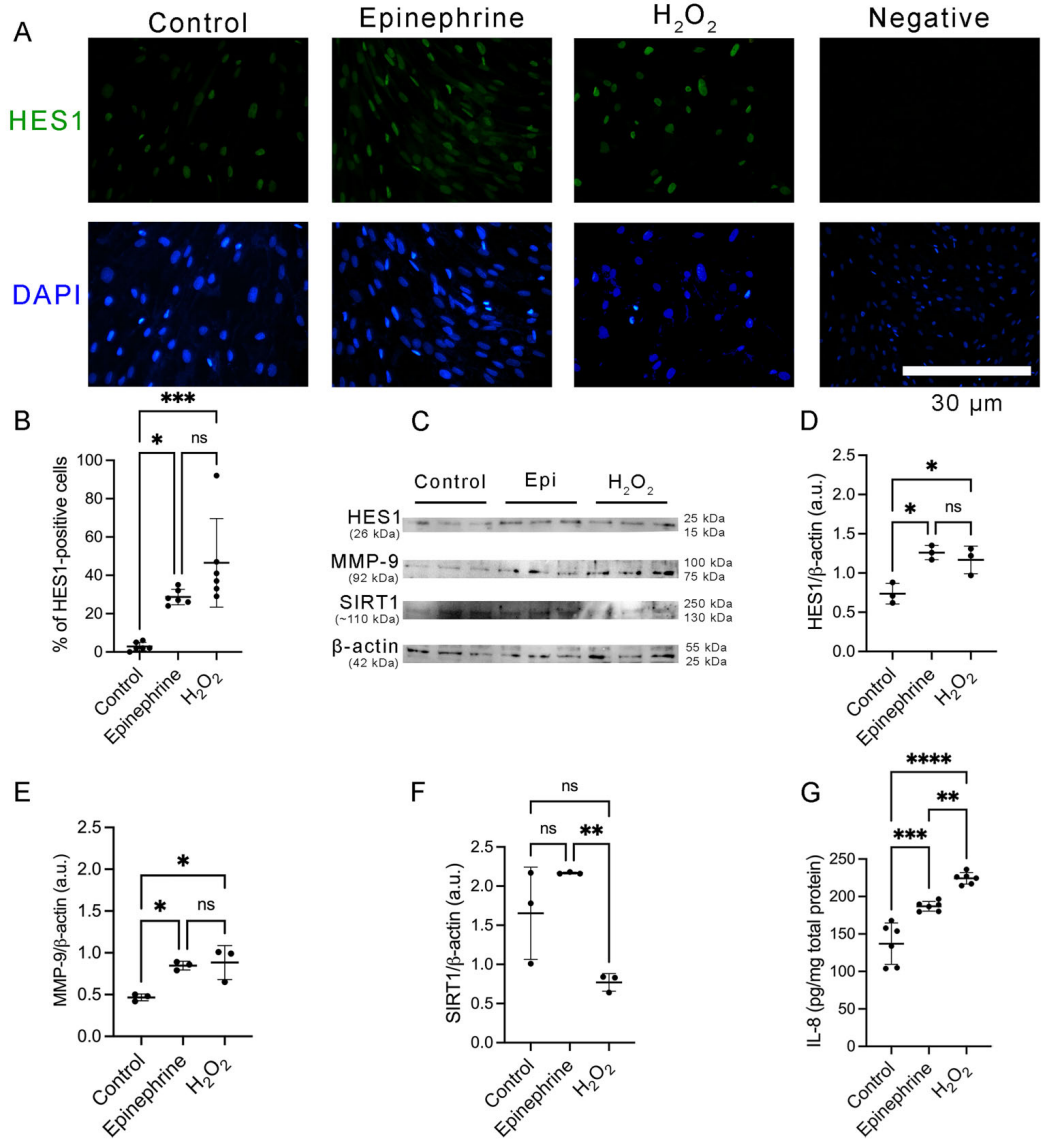
High epinephrine levels promoted Hes1-positive senescent human dermal fibroblasts and augmented the molecular markers of senescent human dermal fibroblasts. **A**, Representative immunofluorescence microscopy images of human dermal fibroblasts stained with an antibody against notch target gene hairy and enhancer of split 1 (HES1) (green) (n=6, two independent experiments in triplicate). Nuclei were stained with DAPI (blue). Scale bar=30 µm. Primary antibody was omitted in negative control. **B**, Percentage of HES1-positive cells normalized by number of DAPI-stained nuclei. **C**, Representative images of immunoblotting for notch target gene hairy and enhancer of split 1 (HES1), matrix metaloproteinase-9 (MMP-9), silent information regulator sirtuin 1 (SIRT1), and β-actin. Protein levels of HES1 (**D**), MMP-9 (**E**), and SIRT1 (**F**) normalized to β-actin using western blot analysis, expressed as arbitrary units (a.u.). Protein levels of interleukin-8 (IL-8) (**G**) measured by ELISA assay, expressed as pg/mg of total protein. Data are reported as means±SD (n=6, two independent experiments in triplicate). Each dot represents a pool of three wells per group. *P<0.05, **P<0.01, ***P<0.001, ****P<0.0001; one-way ANOVA with Bonferroni's post-test; ns: not significant. Epi: epinephrine, H_2_O_2_: hydrogen peroxide.

To evaluate if repeated stress could induce premature senescence in dermal fibroblasts, human dermal fibroblasts were treated with high dose epinephrine to simulate chronically stressed individuals (11), following the SIPS protocol (Figure S1). The SIPS model with hydrogen peroxide (H_2_O_2_) was used as a positive control. The 3-(4,5-dimethyl-2-thiazolyl)-2,5-diphenyl-2H-tetrazolium bromide (MTT) assay showed that administering epinephrine or H_2_O_2_ did not alter the cell viability compared to control (medium only) (Figure 1A) (F (2, 15)=1.679, P=0.2197). The senescence-associated β-galactosidase (SA-β-gal) staining was increased in human dermal fibroblasts treated with epinephrine or H_2_O_2_ compared to control (Figure 1B). However, no significant differences in telomere length were observed between human dermal fibroblasts treated with epinephrine, H_2_O_2_ or medium only (Figure 1C) (F (2, 15)=0.6337, P=0.5443). qRT-PCR analysis demonstrated that mRNA levels of p53 were higher in the fibroblasts treated with epinephrine or H_2_O_2_ than in the fibroblasts treated with medium only (Figure 1D) (F (2, 15)=21.84, P<0.0001). There was no significant difference in the percentage of apoptotic cells (annexin V+ cells) and protein levels of PCNA between human dermal fibroblasts treated with epinephrine, H_2_O_2_, or medium only (Supplementary Figure S2A-C) (F (2, 15)=0.7711, P=0.4800). After treatment with epinephrine or H_2_O_2_, human dermal fibroblasts showed a flattened and enlarged senescent phenotype (Supplementary Figure S2D). Moreover, human dermal fibroblasts treated with epinephrine or H_2_O_2_ showed higher ROS production compared to control (Figure 1E) (F (2, 15)=57.65, P<0.0001). To confirm the induction of premature senescence by a high epinephrine level, the expression of additional senescence associated biomarker was evaluated. HES1 was identified as a senescence marker in human dermal fibroblasts (5). Immunofluorescence analysis indicated that fibroblasts expressing HES1 were augmented in fibroblasts incubated with epinephrine or H_2_O_2_ compared to control (Figure 2A). The percentage of HES1-positive cells was higher in the epinephrine- or H_2_O_2_-treated cells compared to control (Figure 2B) (F (3, 32)=44.80, P<0.0001). Western blot analysis confirmed that the administration of epinephrine or H_2_O_2_ significantly increased the protein levels of HES1 in the human dermal fibroblasts compared to control (Figure 2C and D) (F (2, 6)=12.40, P=0.0074). In addition, the administration of epinephrine or H_2_O_2_ increased the protein levels of MMP-9 compared to control (Figure 2C and E) (F (2, 6)=10.57, P=0.0108). Moreover, an ELISA assay demonstrated that protein levels of IL-8 were significantly augmented in epinephrine- or H_2_O_2_-treated fibroblasts compared to control (Figure 2G) (F (2, 15)=39.89, P<0.0001). We also investigated if stress-stimulated catecholamines could attenuate the expression of SIRT1 that is strongly associated with a longer lifespan (7). Surprisingly, fibroblasts treated with H_2_O_2_ only presented a reduction of protein levels of SIRT1 compared to control estimated by western blot analysis (Figure 2C and F) (F (2, 6)=12.43, P=0.0073). These results confirmed that a high concentration of epinephrine, similar to chronically stressed individuals, can induce a senescent phenotype in human dermal fibroblasts.

### Stress-induced premature senescence worsened wound closure of aged human skin *ex vivo*

**Figure 3:**
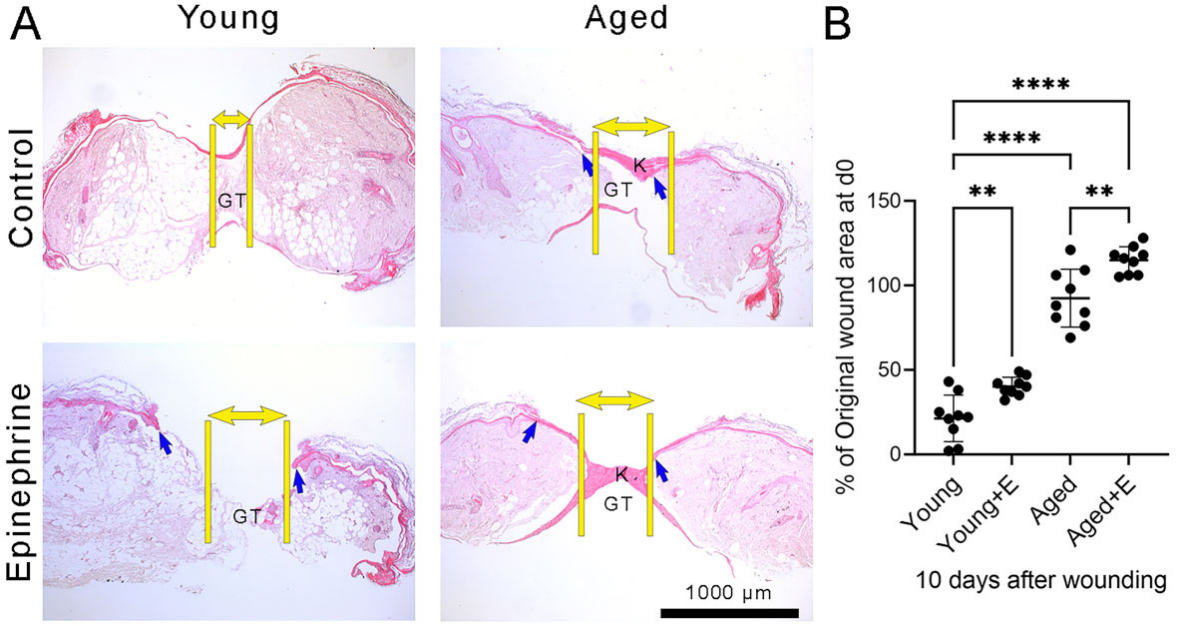
High epinephrine (E) levels aggravated the impairment of wound closure in aged human skin *ex vivo*. **A**, Representative images of the wound area in young and aged human skin stained with hematoxylin and eosin. Scale bar=1000 µm. Yellow lines and double arrows show the edge of the skin wound and blue arrows show the tip of the epidermal tongue. GT: granulation tissue; K: keratinization. **B**, Measurement of wound closure in young and aged human skin *ex vivo*, reported as percentage of original wound area at day 0 (d0). Data are reported as means±SD (n=9, three independent experiments in triplicate). **P<0.01, ****P<0.0001; one-way ANOVA with Bonferroni's post-test.

Subsequently, it was investigated to see if repeated stress would worsen the closure of cutaneous wounds in healthy young and aged humans. Explants of human skin from healthy young and aged subjects were cultured in high epinephrine levels following the SIPS protocol *ex vivo*. Ten days after wounding, the skin wounds of young humans cultivated with medium only exhibited complete re-epithelization (the formation of a new epidermis) and granulation tissue formation (the deposition of new extracellular matrix components in the wound bed) (Figure 3A). However, treatment with a high dose of epinephrine compromised the re-epithelialization and granulation tissue formation in young human skin (Figure 3A). Aged human skin incubated with medium only showed reduced re-epithelialization and granulation tissue formation *ex vivo* (Figure 3A). When treated with epinephrine, aged human skin wounds did not exhibit any granulation tissue formation and partial re-epithelization (Figure 3A). The measurement of wound closure demonstrated that aged human skin treated with medium only presented an impairment of wound closure compared to young human skin treated with medium only (Figure 3B) (F (3, 32)=119.5, P<0.0001). However, the administration of a high epinephrine dose worsened the wound closure of both young and aged human skin compared to controls (young or aged human skin with medium only) (Figure 3B) (F (3, 32)=119.5, P<0.0001).

**Figure 4:**
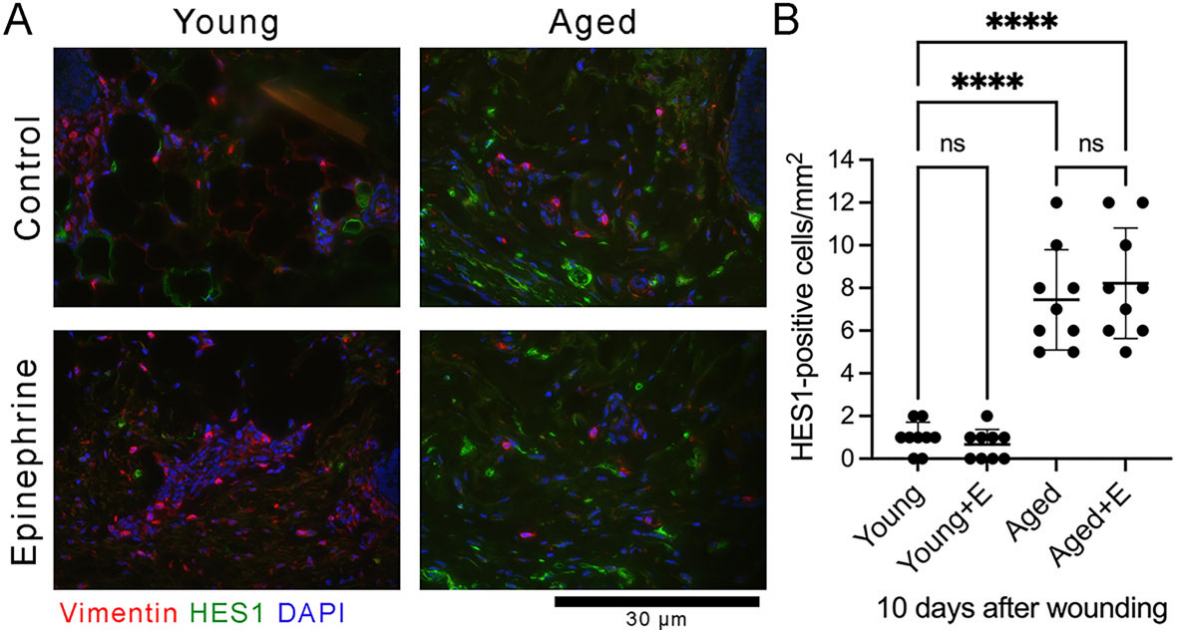
High epinephrine (E) levels increased the number of notch target gene hairy and enhancer of split 1 (HES1)-positive senescent dermal cells in human skin *ex vivo*. **A**, Representative immunofluorescence microscopy images of dermal cells stained with antibodies against HES1 (green) and vimentin (red) in young and aged human skin *ex vivo*. Nuclei were stained with DAPI (blue). Scale bar=30 µm. **B**, Number of cells positive to HES1 in young and aged human skin *ex vivo.* Data are reported as means±SD (n=9, three independent experiments in triplicate). ****P<0.0001; one-way ANOVA with Bonferroni's post-test; ns: not significant.

The expression of senescence biomarker in wound bed of human skin was evaluated using immunofluorescence analysis for HES1. A few HES1-positive cells were observed in the wound bed of young human skin treated with medium only at 10 days after wounding (Figure 4A and B) (F (3, 32)=44.80, P<0.0001). Compared to young skin, more HES1-positive cells were seen in the wound bed of aged human skin treated with medium only at 10 days after wounding (Figure 4A and B) (F (3, 32)=44.80, P<0.0001). The administration of a high epinephrine dose increased the expression of HES1 in the wound bed of aged human skin compared to control (young human skin with medium only) at 10 days after wounding (Figure 4A and B) (F (3, 32)=44.80, P<0.0001). No double cells that were positive for vimentin and HES1 were observed (Figure 4A). These data showed that a high concentration of epinephrine, similar to that associated with repeated stress *in vivo*, worsened wound closure and increased the expression of HES1 in aged human skin *ex vivo*.

## Discussion

Several previous studies demonstrate that repeated stress can impact biological aging in humans and mice. In humans, stress-induced glucocorticoid secretion reduces telomerase activity or directly damages telomeres (10). In mice, chronic psychological stress shortens the lifespan and augments the expression of senescence markers (such as p53, p21, and p16^INK4a^) and SASP in bone marrow cells, liver, and spleen (22-25). Moreover, repeated stress speeds up the signs of skin aging in mice and *ex vivo* human skin (11-14). Stress-stimulated premature senescence can increase the risk of cognitive decline, dementia, and Alzheimer's disease in elderly humans (26). *In vitro,* senescence of dermal fibroblasts can be induced using a classical experimental model for cellular aging called SIPS. In the SIPS model, cellular aging can be accelerated by treating fibroblast cultures with sublethal doses of H_2_O_2_ with a long-term exposure (27). During consecutive sub-cultures, H_2_O_2_-stimulated oxidative damage leads to irreversible molecular changes similar to those associated with aging, such as cell growth arrest, SA-β-gal expression, a reduced apoptosis rate, and a flattened and enlarged morphology (27,28). In addition, senescent fibroblasts also express an increased expression of HES1, which is a biomarker of notch activation, p21, p53, and p16 (5,29). In our study, human dermal fibroblasts exposed to a non-lethal dose of epinephrine following the SIPS model showed several senescence markers *in vitro*, as evidenced in H_2_O_2_-treated cells. Thus, our findings suggest that repeated stress may accelerate the acquisition of senescence in human dermal fibroblasts, which may contribute to skin aging, as previously described (11-14).

Fibroblasts are crucial to dermal reconstruction during skin wound healing, promoting collagen deposition and wound contraction. However, senescent fibroblasts increase the release of several pro-inflammatory factors and proteases, reduce the production of growth factors, and are resistant to apoptosis or removal by immune system cells (5). Senescent fibroblasts acquire a phenotype that is referred to as SASP. The chronic inflammation and proteolytic microenvironment induced by SASP fibroblasts drives an impairment of skin wound healing (30). In our study, high epinephrine levels, similar to those that occur during repeated stress, promoted a pro-inflammatory SASP (higher production of MMP-9 and IL-8) in human dermal fibroblasts, as previously described (6). These data indicate that pro-inflammatory SASP in dermal fibroblasts might be involved in the stress-promoted alterations in human skin.

The natural process of skin aging leads to a delay in the cutaneous wound healing due to a reduction of cell activity (2). Our study was the first to report that skin wound healing in aged humans can be significantly worsened under stressful conditions. Furthermore, wounds in young human skin were also compromised by the treatment with high epinephrine doses, a finding in line with previous studies (16,31,32). In aged human skin, treatment with a high epinephrine dose increased the expression of HES1. This result indicated that stress-induced premature senescence in human dermal fibroblasts likely contributed to the worsening of the skin healing process in aged human skin. These findings are in line with a previous study that observed a delay in skin wound healing of young mice treated with senescent cells (33). We also observed a higher production of ROS in human dermal fibroblasts incubated with a high epinephrine dose. Previous studies have shown that stress-stimulated ROS production is directly associated with signs of aging in *ex vivo* human skin and dermal fibroblasts (11,13,14). Thus, we suggest that stress-induced ROS production might trigger premature senescence and pro-inflammatory SASP in human dermal fibroblasts, which lead to an impaired closure of skin wounds in aged humans.

Sirtuins are nicotinamide adenine dinucleotide-dependent class III histone deacetylases that are largely conserved during evolution (7). Sirtuins are important in the regulation of a broad variety of cellular processes such as metabolism, autophagy, DNA repair, and aging (34). The sirtuin family has seven members (SIRT1-7), but SIRT1 is the sirtuin that is widely involved in the regulation of cellular senescence and organism longevity (34). In dermal fibroblasts, the oxidative damage induced by ultraviolet radiation reduces SIRT1 levels, resulting in the activation of p53 and promoting cellular senescence (35). In this study, high epinephrine doses did not decrease SIRT1 levels in human dermal fibroblasts, indicating that SIRT1 is not involved in the induction of cellular senescence in stressful conditions.

The limitations of this study are mainly associated with a lack of telomere shortening in the SIPS model. Telomeres are a DNA-protein structure at the end of chromosomes that shorten as cells divide due to an end replication problem (36). The oxidative damage or glucocorticoids induced by psychological stress can induce telomere shortening through the reduction of telomerase activity, which is a specialized enzyme responsible for elongating telomeres (10,37). In human dermal fibroblasts, stress with SIPS-inducing concentrations of H_2_O_2_ leads to the loss of cell proliferation, and the shortening of telomeres cannot be observed. In the model studied here, the time interval may have been insufficient to detect telomere shortening, the process of which is cumulative and long-term.

In conclusion, stress-related high epinephrine levels induced premature senescence in human dermal fibroblasts during the skin healing process. *In vitro*, human dermal fibroblasts subjected to high epinephrine levels exhibited a higher expression of SA-β-gal, HES1, and p53 and irreversible proliferation arrest that confirmed a senescent phenotype. *Ex vivo*, stress-related high epinephrine levels disrupted the wound closure of aged human skin through the induction a pro-inflammatory SASP phenotype in senescent fibroblasts. The excessive production of ROS may be involved in the adverse effect of repeated stress on cellular senescence during skin wound healing in aged humans *ex vivo*. These findings can be considered by physicians during the treatment of skin wounds in aged patients under stressful conditions.

## Supplementary Material

Click here to view [pdf].
