# Supplementary material for: Stress-stimulated epinephrine induces premature senescence in dermal fibroblasts and contributes to impaired skin wound healing
Source: Braz J Med Biol Res. 2025 Jun 16;58:e14472. doi: 10.1590/1414-431X2025e14472 (PMC12172154; doi:10.1590/1414-431X2025e14472)
Supplement: Supplementary file 1 [file 1414-431X-bjmbr-58-e14472-suppl.pdf]

Figure S1. Experimental design. **A**, Human dermal fibroblasts model. **B**, Normal human skin explant model.

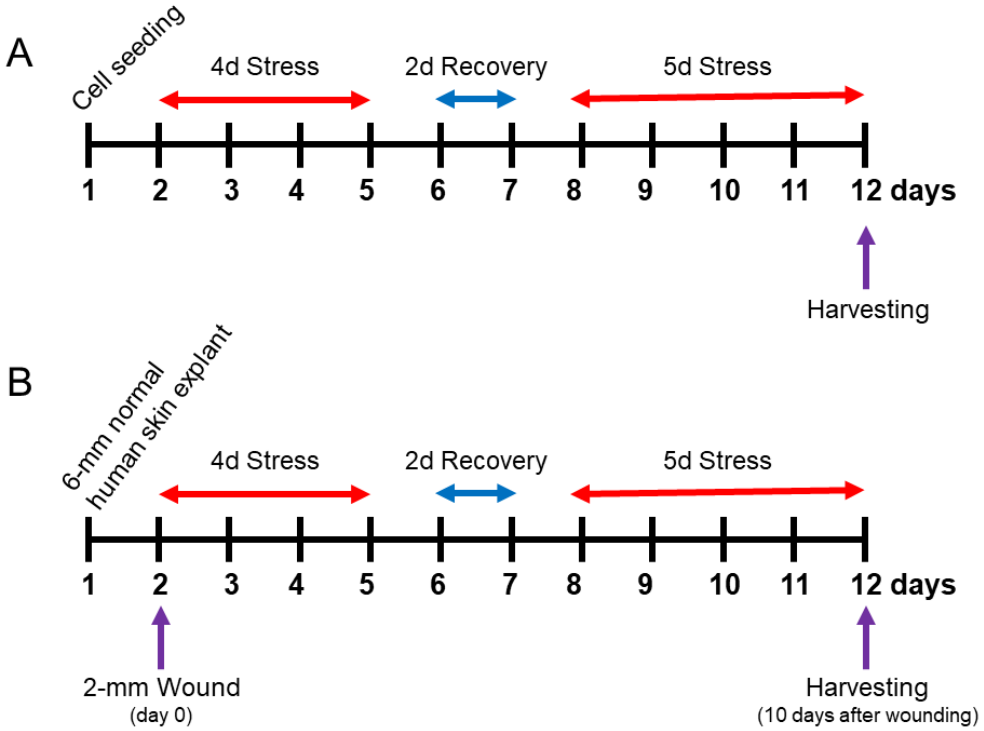

Figure S2. Characterization of stress-induced premature senescent (SIPS) fibroblasts. **A**, Flow cytometry analysis showing the percentage of total apoptotic cells (red square) in unstained, control (medium only), and epinephrine-treated cells. **B**, Protein levels of proliferating cellular nuclear antigen (PCNA) normalized to β-actin using western blot analysis in arbitrary units (a.u.). **C**, Representative images of immunoblotting for PCNA and β-actin. **D**, Representative photomicrographs of epinephrine-treated and SIPS cells. Scale bar=20 μm. Data are reported as means±SD (n=6, two independent experiments in triplicate). ns: not significant; one-way ANOVA with Bonferroni's post-test. H_2O_2: hydrogen peroxide.

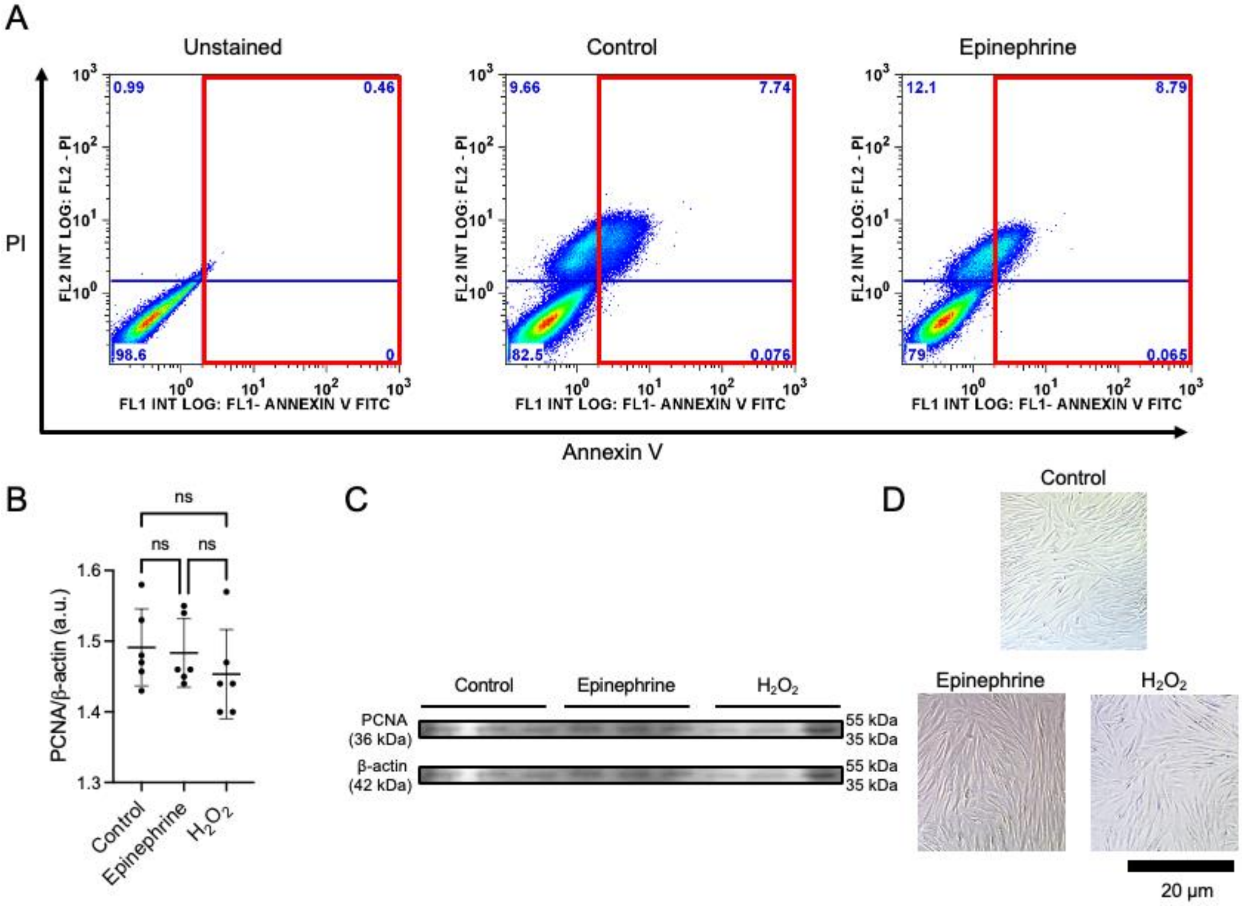

Table S1. Antibodies used in immunocytochemistry, immunohistochemistry, and western blot.

Antibody	Target	Produced in	Dilution	Clone	Dye/enzyme	Company	Catalog number
β-actin	Rat, Mouse Rabbit, Pig, Human	Mouse	1:5000	AC-15	–	Sigma-Aldrich, Inc., USA	A5441
Notch target gene hairy and enhancer of split 1 (HES1)	Human, Mouse, Rat	Rabbit	1:100	SC06-21	–	Invitrogen, USA	MA5-32258
Matrix metalloproteinase-9 (MMP-9)	Human, Mouse	Rabbit	1:2000	Polyclonal	–	Invitrogen, USA	PA5-13199
Proliferating cellular nuclear antigen (PCNA)	Human	Mouse	1:200	clone PC10	–	DAKO Agilent, USA	M0879
Silent information regulator sirtuin 1 (SIRT1)	Human, Mouse, Dog	Rabbit	1:1000	Polyclonal	–	Bioss Antibodies, USA	bs-2257R
Vimentin	Human, Mouse, Rat	Mouse	1:200	V9	–	Santa Cruz Biotechnology, Inc., USA	sc-6290
Secondary antibodies	Anti-rabbit	Goat	1:500	–	Alexa Fluor 488	Invitrogen, USA	A-11008
	Anti-rabbit	Goat	1:1000	–	Horseradish peroxidase	Santa Cruz Biotechnology, Inc., USA	sc-2004
	Anti-mouse	EnVision+ System - HRP Labelled Polymer	1:200	–	Horseradish peroxidase	DAKO Agilent, USA	K4001
	Anti-mouse	Goat	1:800	–	Texa red	Invitrogen, USA	T6390

Table S2. Primers used in quantitative reverse transcription polymerase chain reaction (qRT-PCR) and expression of telomeres.

Name	Target	Forward primer	Reverse Primer	Company
β-globin	Human	5'-GCTTCTGACACAACTGTGTTCACTAGC-3'	5'-CACCAACTTCATCCACGTTCACC-3'	Invitrogen, USA
Glyceraldehyde-3-phosphate dehydrogenase (GAPDH)	Human	5'-CAGGGCTGCTTTTAACTCTGG-3'	5'-TGGGTGGAATCATATTGGAACA-3'	Integrated DNA Technologies IDT, USA
p53	Human	5'-AGGGATGTTTGGGAGATGTAAG-3'	5'-CCTGGTTAGTACGGTGAAGTG-3'	Integrated DNA Technologies IDT, USA
Telomere	Human	5'-CGCTTTGTTTGGGTTTG GGTTTGGGTTTGGGTTTGGGTT-3'	5'-GGCTTGCCTTACCCTTACCCTT ACCCTTACCCTTACCCT-3'	Invitrogen, USA
